# Diffusion weighted imaging as a biomarker of retinoic acid induced myelomeningocele

**DOI:** 10.1371/journal.pone.0253583

**Published:** 2021-06-30

**Authors:** Nathan Maassel, James Farrelly, Daniel Coman, Mollie Freedman-Weiss, Samantha Ahle, Sarah Ullrich, Nicholas Yung, Fahmeed Hyder, David Stitelman

**Affiliations:** 1 Department of Surgery, Yale School of Medicine, New Haven, Connecticut, United States of America; 2 Department of Radiology and Biomedical Imaging, Yale School of Medicine, New Haven, Connecticut, United States of America; 3 Department of Biomedical Engineering, Yale School of Engineering & Applied Science, New Haven, Connecticut, United States of America; 4 Division of Pediatric Surgery, Department of Surgery, Yale School of Medicine, New Haven, Connecticut, United States of America; Henry Ford Health System, UNITED STATES

## Abstract

Neural tube defects are a common congenital anomaly involving incomplete closure of the spinal cord. Myelomeningocele (MMC) is a severe form in which there is complete exposure of neural tissue with a lack of skin, soft tissue, or bony covering to protect the spinal cord. The all-trans retinoic acid (ATRA) induced rat model of (MMC) is a reproducible, cost-effective means of studying this disease; however, there are limited modalities to objectively quantify disease severity, or potential benefits from experimental therapies. We sought to determine the feasibility of detecting differences between MMC and wild type (WT) rat fetuses using diffusion magnetic resonance imaging techniques (MRI). Rat dams were gavage-fed ATRA to produce MMC defects in fetuses, which were surgically delivered prior to term. Average diffusion coefficient (ADC) and fractional anisotropy (FA) maps were obtained for each fetus. Brain volumes and two anatomically defined brain length measurements (D1 and D2) were significantly decreased in MMC compared to WT. Mean ADC signal was significantly increased in MMC compared to WT, but no difference was found for FA signal. In summary, ADC and brain measurements were significantly different between WT and MMC rat fetuses. ADC could be a useful complementary imaging biomarker to current histopathologic analysis of MMC models, and potentially expedite therapeutic research for this disease.

## Introduction

Spina bifida is a type of fetal malformation characterized by failure of neural tube closure during gestational development. The incidence of neural tube defects (NTD) is variable, but estimates range from 1–7 per 1000 live births and differ by region, ethnicity, and sex [1–3]. Myelomeningocele (MMC) is the most common and severe NTD, resulting in complete exposure of spinal cord and meninges at birth. The majority of MMC patients are also born with a Chiari II malformation, defined by downward displacement of their cerebellum and medulla, which can cause poor CSF outflow and hydrocephalus. The increased intracranial pressure can lead to a host of other neurologic sequelae including cognitive dysfunction, dysphagia, and difficulty breathing in these patients, which often necessitates shunt placement [4, 5]. Exposure of the spinal cord to mechanical and chemical stressors during gestation leads to permanent neural injury characterized by urinary and fecal incontinence and complete paralysis of the lower extremities in most patients. MMC treatment options include pre- or post-natal surgery to correct the skin defect in addition to post-natal ventricular shunting. Neonatal mortality remains high for MMC babies at around 10%, and while many MMC patients go on to complete high school and attend college, only about 50% of survivors are capable of living independently as adults [6, 7].

Early *in utero* treatment for MMC remains an ongoing area of research, but, as there is no reliable objective way to analyze disease severity or treatment effectiveness in early gestation, an imaging biomarker that correlates with known histopathological findings would be critical. The all-trans retinoic acid (ATRA) induced MMC rat model is a reproducible and reliable model for testing novel therapies [8–10]. Although ATRA can produce MMC defects in a majority of exposed fetuses, it is difficult to determine structural and functional differences between prenatal and postnatal MMC and wild type (WT) pups. Magnetic Resonance Imaging (MRI) has been used to quantify the severity of Chiari malformations, and high-resolution computed tomography (CT) has been used to characterize vertebral body defects, but MRI and/or CT data has not been particularly useful in the quantitative comparison of defects themselves [11, 12]. This is important especially for experiments where defect coverage/correction in utero is the method of intervention. Currently, post-mortem histology is the primary way to demonstrate presumed potential benefit from prenatal therapies designed to cover the MMC defect prior to birth; however, histology requires euthanasia of the specimens and does not provide sufficient insight into structural or functional correlates. Ideally, an imaging biomarker test would objectively quantify neurologic injury in fetuses to more reliably monitor the results of a corrective intervention.

Diffusion Weighted Imaging (DWI) and Diffusion Tensor Imaging (DTI) are structural MRI techniques that quantify water diffusion in a specific region of tissue. These techniques have been previously used to evaluate spinal cord injury in various animal and human studies [13–18]. The apparent diffusion coefficient (ADC) is a DWI measurement that specifies the magnitude of water diffusion over a specific region, whereas fractional anisotropy (FA) is a DTI measurement that specifies the degree of directional diffusion of water [16]. Although ADC and FA are typically not mapped at histological resolution, they can provide cellular information since ADC can reflect degree of cellularity and FA can represent fiber directionality. We sought to investigate differences in ADC, FA and volumetric data of the central nervous system between WT and MMC groups as a proof of concept for potential future assessment in therapeutic studies.

## Materials and methods

### Retinoic acid induced MMC model

MMC rat fetuses were generated using previously described methodology [8–10, 19–23]. In short, time-dated pregnant Sprague-Dawley dams (Charles River Laboratories; Wilmington, MA) were gavage-fed 1cc of 40 mg/kg, 97% pure ATRA (Across Organics; Morris Plains, NJ) dissolved in olive oil (Whole Foods Market; Austin, TX) on day 10 of gestation. Dams underwent routine daily monitoring for hydration status, activity level, and maintenance of proper nutritional status. WT fetuses were generated using time-dated pregnant dams that did not undergo ATRA gavage. Two dams were used for MMC and two for WT. All animal use was in accordance with the guidelines of Yale University’s Institutional Animal Care and Use Committee. Approval was also granted from this same committee after review by the Yale Office of Animal Research Support (protocol #: 2017–11632).

### MRI of brain and spinal cord

On gestational day 17, WT and MMC dams were euthanized via CO2 followed by cervical dislocation. The fetuses (6 from MMC, 6 from WT) were then harvested and placed into a custom-built MRI-compatible tube filled with Fluorinert—an MRI susceptibility-matching fluid (Sigma-Aldrich). MRI images were obtained on an 11.7 T horizontal bore scanner (Bruker, Billerica, MA) with a bore size of 9 cm and maximum gradient strength of 400 mT/m, using a custom-made ^1^H radio frequency volume coil (4 cm diameter). The DTI experiments were performed using a spin-echo diffusion-weighted sequence with 4 shots, a diffusion gradient of 6 ms and a delay between the two diffusion gradients of 12 ms. 16 contiguous slices of 0.5 mm thickness were acquired at a resolution of 180 × 110 with a field of view of 36mm × 22mm using a repetition time (TR) of 4s, an echo time (TE) of 21 ms and 12 averages. 20 different images were acquired for each slice, 15 corresponding to various non-collinear diffusion gradient directions with b = 1,000 s mm^−2^ and 5 with no diffusion gradients. The total acquisition time was 4.5 hours. Although data was acquired at high field (11.7T), no image correction was necessary. FA and ADC images were generated from the diffusion-weighted spin-echo images in BioImage Suite (http://www.bioimagesuite.org/).

**Fig 1:**
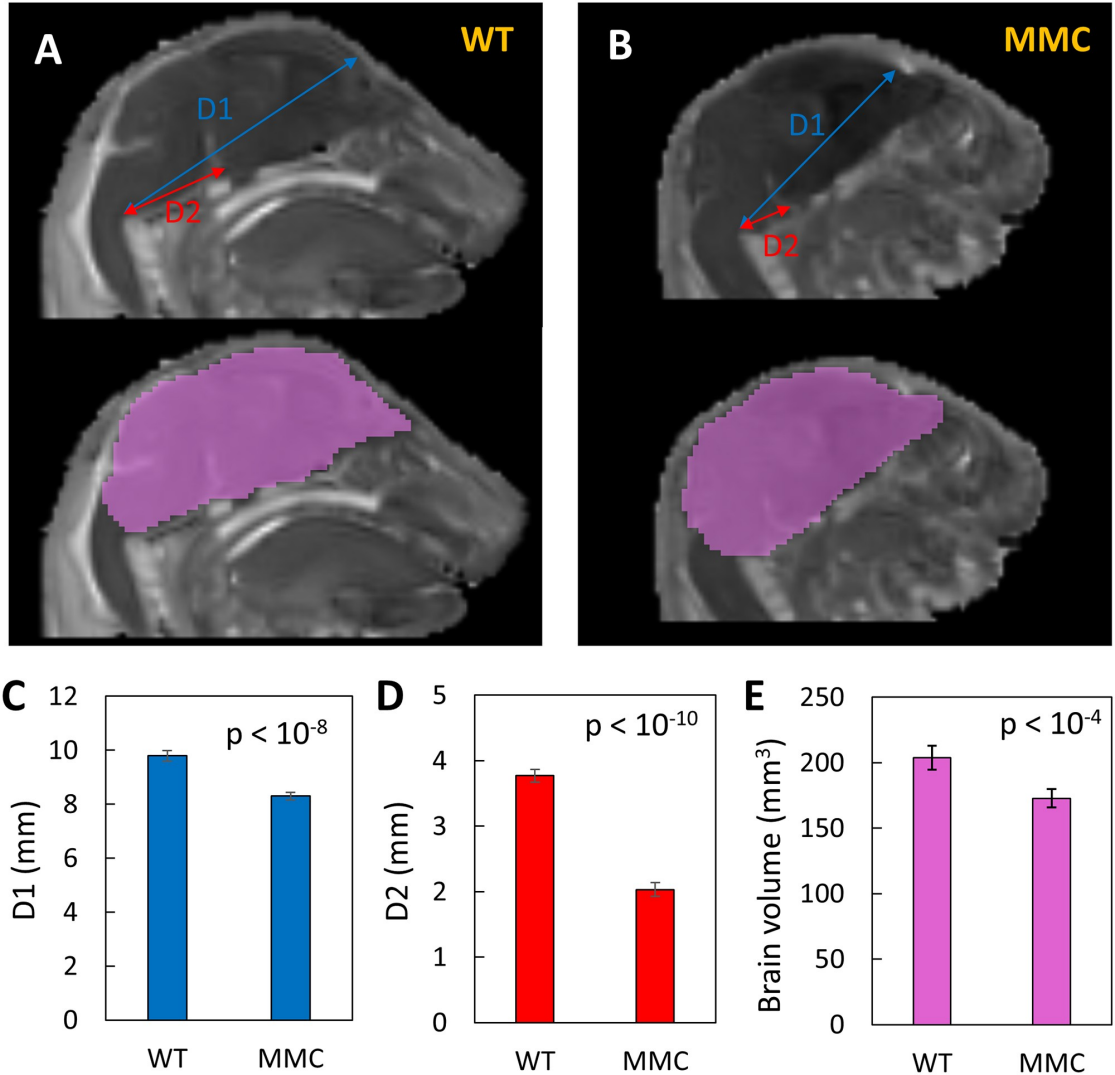
Measurements of brain size for the MMC and WT groups. Examples of D1 and D2 distances (top) and brain volume (bottom) in WT (**A**) and MMC (**B**) groups. D1 and D2 represent the distance from the lateral reticular nucleus to the frontal association cortex or the interpeduncular nucleus, respectively. The brain volume was determined by manually drawing a ROI over the entire brain using the ADC map which provided the best image contrast, followed by calculation of the ROI volume. The distances D1 (**C**) and D2 (**D**), and the brain volume (**E**) were significantly smaller in the MMC compared to the WT group.

**Fig 2:**
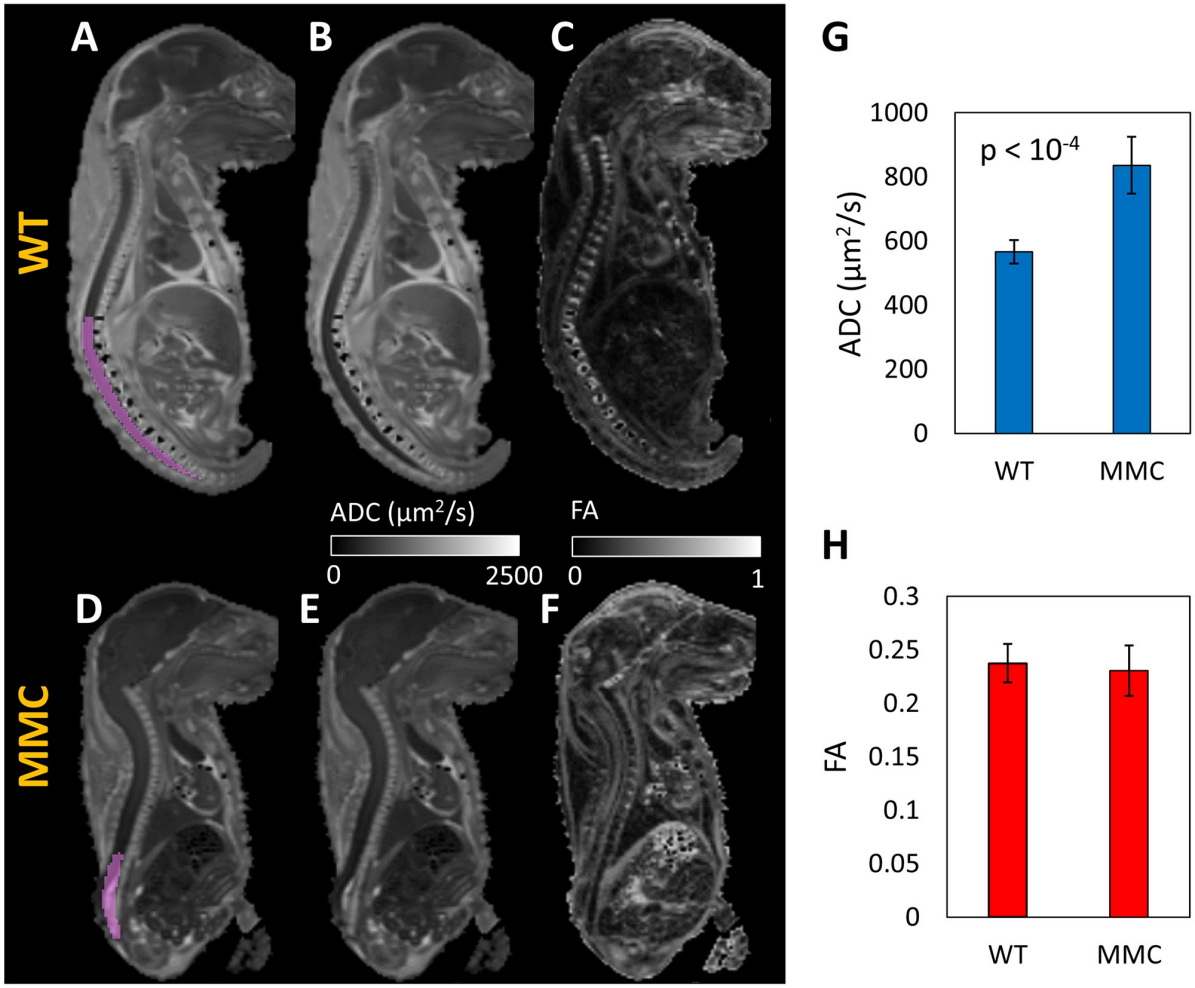
FA and ADC in the spinal cord measured over a selected lumbosacral ROI. Examples of selected spinal cord ROI (**A, D**), ADC maps (**B, E**) and FA maps (**C, F**), in WT and MMC fetuses. Statistically higher ADC (p<0.0001) was measured in the MMC group (**G**). No significant differences were observed in the FA values when comparing the MMC and WT groups (**H**).

The brain distances D1 and D2 were standardized using anatomic landmarks. D1 represents the distance from the lateral reticular nucleus to the frontal association cortex, while D2 represents the distance from the lateral reticular nucleus to the interpeduncular nucleus (Fig 1A and 1B, top). The brain volume was determined by manually drawing a region-of-interest (ROI) over the entire brain using the ADC map which provided the best image contrast, followed by calculation of the ROI volume (Fig 1A and 1B, bottom). The spinal cord was analyzed using a manually selected ROI over the lower spinal cord (Fig 2A and 2D), for which the average ADC and FA were calculated. Manual ROI determination was chosen due to inaccurate delineation when using an intensity-standardized threshold approach.

### Statistical analysis

Averages and standard deviations were calculated for brain distances D1 and D2, brain volume, and spinal cord ADC and FA in BioImage Suite. Group comparisons were performed using a two-tailed Student’s t-test in BioImage Suite. Statistical significance was considered p<0.05.

## Results

The brain size comparison between the MMC (n = 6) and WT (n = 6) groups used three separate measurements: brain volume and two distances D1 and D2, representing the distance from the lateral reticular nucleus to the frontal association cortex or the interpeduncular nucleus, respectively (Fig 1). Average distances D1 and D2 measured in WT specimens were significantly larger than MMC (9.79±0.19 mm, D1 and 3.77±0.10 mm, D2 for WT vs 8.30±0.14 mm, D1 and 2.03±0.10 mm, D2 for MMC, p<0.0001) (Fig 1 and Table 1). In addition, brain volume measurements for the WT group were significantly larger than those of the MMC group (204±9 mm^3^ for WT vs 173±7 mm^3^ for MMC, p<0.0001) (Fig 1 and Table 1).

**Table 1:** Wild type vs myelomeningocele rat fetus brain, spine, FA, and ADC measurements.

	WT	std	MMC	std	p value
**Brain Length D1, mm**	9.79	0.19	8.3	0.14	<0.0001
**Brain Length D2, mm**	3.77	0.1	2.03	0.1	<0.0001
**Brain Volume, mm**^**3**^	204	9	173	7	<0.0001
**Spinal ROI Volume, mm**^**3**^	11.7	1	11	0.6	0.1100
**FA**	0.24	0.02	0.23	0.02	0.5400
**ADC, μm**^**2**^**/s**	566	37	836	89	<0.0001

The average ADC signal over the selected ROI in the spinal cord (Fig 2) was significantly higher in the MMC group when compared to the WT (836±89 μm^2^/s for MMC vs 566±37 μm^2^/s for WT, p<0.0001). There were no statistically significant differences (p = 0.54) in spinal cord FA between the two groups (Fig 2). The average volume of the selected ROI of the spinal cord used for ADC and FA analysis was 11.7±1.0 mm^3^ for the WT group and 11.0±0.6 mm^3^ for the MMC group (Table 1). The statistical comparison between WT and MMC demonstrates similar ROI volumes for both groups.

## Discussion

This study investigated a novel method for comparing the structure of ATRA-induced MMC rat brains and spinal cords with those of WT rats. MMC fetuses were found to have smaller brain length and volume in addition to higher ADC signal in the spinal cord compared to WT. There was no significant difference in the FA signal between the two groups. ADC signal and brain dimensions could be useful adjuncts for quantifying the effects of therapeutic interventions in the rat MMC model and may expedite clinical translation. The fact that ADC is higher in MMC with FA being indifferent from WT is significant. These data suggest that MMC spinal cords might have reduced cellularity but similar directionality of cellular connections when compared to WT specimens.

The ability to objectively quantify injury in animal models for MMC without causing harm to the fetus is essential to evaluating therapeutic strategies developed in the laboratory setting. Much of the current literature about in-utero treatment of MMC focuses on coverage or water-tight repair of the spina bifida defect prior to term and appropriately displays histological analyses to quantify success [21–27]. After development of a compelling novel MMC therapy, meaningful functional outcomes would provide a clear step towards human clinical translation; however, functional neurological evaluation is especially challenging in a sick small-animal model such as the ATRA-induced rat model. There are several composite scoring systems that exist for the assessment of neurologic function in rats, but these methods require survival to term, continuous monitoring, and are more commonly used in adult models for stroke and spinal cord injury [28, 29]. For this reason, utilization of non-invasive diffusion-based MRI to complement histologic evidence of defect coverage is extremely useful. Although future *in vivo* DWI studies are needed to establish this *ex vivo* DWI finding, it is anticipated that a 50% increase in ADC for MMC would be detectable even under the most modest MRI scanner situations.

The current understanding of the pathology of spinal cord injury in spina bifida involves a two-hit disease process, whereby a primary error in neural tube development is made significantly worse by secondary injury from chemical and mechanical damage to spinal cord tissue while *in utero* [30, 31]. In human applications of DTI, specific to spina bifida, there have been reports of abnormal white matter development and decreased FA signal, suggesting axonal injury [32]. Though our study found no significant difference in FA, the average ADC was significantly higher in the MMC rat fetuses compared to WT. One possible explanation for an increased ADC in the MMC fetuses is the increased water mobility due to extracellular edema and cell degeneration from the “second hit” in myelomeningocele pathogenesis. Although this is an ongoing area of research, there have been several studies detailing an increase in digestive enzymes within amniotic fluid of rats with MMC, as a possible mechanism for injury [33, 34]. In human spinal cord studies, ADC signal has been shown to increase with age related spinal cord degeneration, explained at least partially by chronic ischemia and natural degenerative changes [16]. This is in contrast to the decrease seen in ADC signal at 24 and 72 hours in acute rat spinal cord injury models [35]. Unlike the acute traumatic pathology of the rat spinal cord injury model, the pathologic timeline of MMC is likely more analogous to a chronic process; however, there is no baseline measurement of ADC or FA in the rat model of MMC for comparison. Apart from toxicity related to exposure in the amniotic environment, human studies of young patients with spina bifida suggest disruption of the sacral plexus as an additional pathophysiologic explanation for paralysis and genitourinary dysfunction in these patients. In a series of 10 patients age 8 to 16 years old, Haakma et al. demonstrated asymmetry and disorganization of the sacral plexus using DTI and fiber tractography [36]. These findings demonstrate the range of additional applications for diffusion MRI techniques that could be applied in the experimental and clinical setting to potentially measure MMC severity or improvement following a therapeutic intervention.

The limitations of this study are its relatively small group size (n = 6 in each group) and single gestational time point for analysis. Additionally, though the MRI data investigated in this study was obtained using a high field pre-clinical scanner (11.7T), we expect that the results would be reproducible at lower magnetic fields (i.e., 1.5T-7T). Water diffusion properties (like ADC and FA) are not field dependent, which makes MRI sequences such as DWI or DTI more translatable. Despite these limitations, the overarching purpose of this study was to pilot structural and anatomical MRI techniques as possible methods for discerning MMC from WT as a step towards non-invasive monitoring of therapeutic success following a pre-natal intervention in the rat model of MMC. As experimental therapies for MMC move toward clinical trials, diffusion MRI may prove useful for post-therapy monitoring in humans as well, however these applications are beyond the scope of this study. Future directions for this line of research include a therapeutic group and a wider range of gestational ages to term (E21).

## Conclusions

Analysis of objective neurologic outcomes in the rat model of MMC is challenging during the early stages of pup development. ADC and brain size measurements using diffusion MRI could provide a non-invasive platform to quantify both severity of injury and objective treatment response in the ATRA-induced rat MMC model. Future studies that analyze the long-term functional neurological outcomes of treated and untreated MMC pups will be crucial for determining the validity of non-invasive, prenatal MRI for measuring prenatal MMC therapy response.

## Supporting information

S1 Dataset Individual animal data with summary statistics and corresponding figures. (XLSX)
